# *RANK* rs1805034 T>C Polymorphism Is Associated with Susceptibility of Esophageal Cancer in a Chinese Population

**DOI:** 10.1371/journal.pone.0101705

**Published:** 2014-07-14

**Authors:** Jun Yin, Liming Wang, Weifeng Tang, Xu Wang, Lu Lv, Aizhong Shao, Yijun Shi, Guowen Ding, Suocheng Chen, Haiyong Gu

**Affiliations:** 1 Department of Cardiothoracic Surgery, Affiliated People's Hospital of Jiangsu University, Zhenjiang, China; 2 Cancer institute, Department of Chemotherapy, Affiliated People's Hospital of Jiangsu University, Zhenjiang, Jiangsu, China; The University of Hong Kong, China

## Abstract

Esophageal cancer remains the sixth leading cause of cancer associated death and eighth most common cancer worldwide. Genetic factors, such as single nucleotide polymorphisms (SNPs), may contribute to the carcinogenesis of esophageal cancer. Here, we conducted a hospital based case-control study to evaluate the genetic susceptibility of functional SNPs on the development of esophageal cancer. A total of 629 esophageal squamous cell carcinoma (ESCC) cases and 686 controls were enrolled for this study. The *OPG* rs3102735 T>C, rs2073618 G>C, *RANK* rs1805034 T>C, *RANKL* rs9533156 T>C and rs2277438 A>G were determined by ligation detection reaction method. Our findings suggested that *RANK* rs1805034 T>C is associated with the susceptibility of ESCC, which is more evident in male and elder (≥63) patients. Our study provides the first evidence that functional polymorphisms *RANK* rs1805034 T>C may be an indicator for individual susceptibility to ESCC. However, further larger studies among different ethnic populations are warranted to verify our conclusion.

## Introduction

Despite recent considerable medical advances, esophageal cancer remains a refractory disease with high morbidity and mortality. Essentially, esophageal cancer is the 6^th^ leading cause of cancer-related mortality and the 8^th^ most common cancer worldwide [1]. There are more than 450,000 patients diagnosed as esophageal cancer worldwide and the incidence is still rising rapidly. Meanwhile, its startling overall 5-year survival rate ranges from 15∼25% [2]. In China, more strikingly, esophageal cancer ranks the 5^th^ most common diagnosed cancer and 4^th^ leading cause of cancer related mortality [3]. Esophageal squamous cell carcinoma (ESCC) is the predominant histological type of esophageal cancer [1]. Although multidisciplinary therapeutic strategy has been recommended, the prognosis is still poor. Tobacco use [4], [5], alcohol consumption [4], [6], low socioeconomic status, poor oral hygiene and nutritional deficiencies [2], [7]–[9] have been identified as risk factors for esophageal cancer. Yet, only a subset of individuals exposed to these risk factors eventually develop esophageal cancer, indicating a pivotal role of genetic factors, such as single nucleotide polymorphisms (SNPs), in the esophageal carcinogenesis.

Recently, the osteoprotegerin (OPG), its binding protein–the receptor activator of NF-κB (RANK) and RANK ligand (RANKL) have been implicated with the pathogenesis of breast cancer [10]. OPG was initially identified from a fetal rat intestine cDNA library [11], which is unique for it only exists as a secreted molecule in contrast to the other membrane-bound cell surface members of tumor necrosis factor receptor (TNF-R) family. RANKL is the OPG binding protein (also named OPG ligand, OPGL) [12], [13], while RANK constitutes the cell surface receptor which responses to OPGL. In numerous rodent models of tumor, RANKL signal is increased through diverse mechanisms [14]. OPG neutralizes RANKL, which leads to a reduced RANKL-RANK interaction [12]. RANKL expression was verified in various tumor types and inflammatory cells associated with tumor [15]–[17]. Elevation in stromal RANKL has been detected at local sites of bone metastasis or multiple myeloma [18], [19], causing enhanced osteoclast activity and bone destruction. In experimental models, RANKL inhibitors reduced tumor-induced osteolysis in various types of cancer [14], reduced bone destruction, skeletal tumor progression, as well as tumor burden [17], [20], [21]. In addition, RANKL-RANK pathway may contribute to the primary tumorigenesis and metastasis independently of its effects on tumor-related osteolysis. Regulated by factors including prolactin and progesterone, RANKL could drive the primary mitogenic response of mammary epithelium and the expansion of mammary stem cells via RANK activation [22]–[24], which may therefore induce mammary cancer by offering a more transformation-susceptible target pool. RANKL may regulate spontaneous mammary tumor formation and metastasis driven by the potent oncogene *Neu* (*ERBB2*). RANKL blockade effectively attenuated the formation of mammary tumors and pulmonary metastasis in the MMTV-Neu transgenic mouse model [25], [26]. Interestingly, OPG may serve as a positive regulator of microvessel formation and may promote neovascularization [27] that is important for tumor progression. OPG overexpression by breast cancer cells enhances orthotopic and osseous tumor growth [28]. In light of all these findings, RANKL/RANK/OPG signaling pathway has emerged as a promising therapeutic target of cancer. Denosumab, a monoclonal antibody against RANKL, has been approved for the treatment of postmenopausal osteoporosis and bone metastasis in breast cancer [29].

Genetic variations in genes encoding RANK, RANKL and OPG were found to affect the rheumatoid arthritis [30], Paget's disease of bone [31], hip osteoporotic fracture [32]. More importantly, in the Caucasian population, a significant association of the SNP rs3102735 (*OPG*) with the susceptibility to develop breast cancer has been reported [10]. The functional significance of RANK/RANKL/OPG signaling pathway and pilot study in breast cancer have led us to investigate the association between the esophageal cancer and SNPs in the genes of *RANK*, *RANKL* and *OPG*. In a hospital-based case-control study, we performed genotyping analyses of the five miRNA SNPs in 629 ESCC cases and 686 controls in a Chinese population.

## Methods

### Ethics Statement

This hospital-based case-control study was approved by the Review Board of Jiangsu University (Zhenjiang, China). All subjects provided written informed consent to be included in the study. We have complied with the World Medical Association Declaration of Helsinki regarding ethical conduct of research involving human subjects and/or animals.

### Study populations

A total of 1,315 participants consisting of 629 esophageal cancer patients and 686 non-cancer controls frequency-matched to the cases with regard to age (±5 years) and sex were enrolled in this study (Table 1). All patients and controls were consecutively recruited from the Affiliated People's Hospital of Jiangsu University and Affiliated Hospital of Jiangsu University (Zhenjiang, China) between October 2008 and December 2010. All cases of esophageal cancer were diagnosed as ESCC histologically. Patients who had cancer history/metastasized cancer or had received chemotherapy/radiotherapy were excluded for the current study.

**Table 1:** Distribution of selected demographic variables and risk factors in ESCC cases and controls.

Variable	Cases (n = 629)		Controls (n = 686)		*p* a
	n	%	n	%	
**Age (years)** mean ± SD	62.85 (±8.13)		62.58 (±7.89)		0.541
**Age (years)**					0.155
<63	310	49.28	365	53.21	
≥63	319	50.72	321	46.79	
**Sex**					0.185
Male	444	70.59	461	67.20	
Female	185	29.41	225	32.80	
**Tobacco use**					**<0.001**
Never	355	56.44	499	72.74	
Ever	274	43.56	187	27.26	
**Alcohol use**					**<0.001**
Never	428	68.04	526	76.68	
Ever	201	31.96	160	23.32	

a Two-sided *χ* ^2^ test and student t test; Bold values are statistically significant (*p*<0.05).

Each subject was personally questioned by experienced interviewers using a questionnaire to obtain information on demographic data (e.g., age, sex) and related risk factors (including tobacco use and alcohol consumption). After the interview, 2-mL samples of venous blood were collected from each subject. “Smokers” subgroup included individuals who smoked one cigarette per day for >1 year. Subjects who consumed ≥3 alcoholic drinks a week for >6 months were subdivided into “alcohol drinkers” category.

### Genomic DNA extraction, SNP selection and Genotyping

Genomic DNA was isolated from peripheral blood using QIAamp DNA Blood Mini Kit (Qiagen, Berlin, Germany) as reported previously [33]. Sample DNA were amplified by PCR according to the manufacturer's protocol. The samples were genotyped using the ligation detection reaction (LDR) method [34] (technical support from the Biowing Applied Biotechnology™, Shanghai, China). Analyses were repeated in 160 random samples (12.17%) with high DNA quality for quality control.

### Statistical Analyses

Variations of demographic characteristics, selected variables, and genotypes of the *OPG* rs3102735 T>C, rs2073618 G>C, *RANK* rs1805034 T>C, *RANKL* rs9533156 T>C and rs2277438 A>G variants between the cases and controls were evaluated using the *χ* ^2^ test. The associations between the five SNPs and risk of ESCC were assessed by computing the ORs and their 95% CIs using logistic regression analyses for crude ORs and adjusted ORs when adjusting for age, sex, tobacco smoking and alcohol drinking status. The Hardy-Weinberg equilibrium (HWE) was tested by a goodness-of-fit *χ* ^2^ test to compare the observed genotype frequencies to the expected ones among the control subjects. All statistical analyses were performed with SAS 9.1.3 (SAS Institute, Cary, NC).

## Results

### Characteristics of the study population

Characteristics of cases and controls included in the study are summarized in Table 1. The cases and controls are adequately matched on age and sex as evaluated by the *χ* ^2^ tests. Notably, significant difference exists on both tobacco smoking and alcohol drinking status between the ESCC patients and the controls (*p*<0.001).

The primary information for five genotyped SNPs was shown in Table 2. In all 1315 samples, the success rate of genotyping was 95.13%, 96.35%, 96.43%, 96.43% and 96.81% for *OPG* rs3102735 C>T, *OPG* rs2073618 G>C, *RANK* rs1805034 T>C, *RANKL* rs9533156 T>C and *RANKL* rs2277438 A>G, respectively. The concordance rates of repeated analyses reached 100%. As for the Minor Allele Frequency (MAF), there was no significant difference between our controls and database of Chinese subjects for all five SNPs. The observed genotype frequencies for *OPG* rs3102735 C>T, *OPG* rs2073618 G>C, *RANK* rs1805034 T>C, *RANKL* rs9533156 T>C and *RANKL* rs2277438 A>G polymorphisms in the controls were in HWE (*p* = 0.191, 0.371, 0.531, 0.488 and 0.700, respectively)(Table 2).

**Table 2:** Primary information for *OPG* rs3102735 T>C, rs2073618 G>C, *RANK* rs1805034 T>C, *RANKL* rs9533156 T>C and rs2277438 A>G polymorphisms.

Genotyped SNPs	*OPG* rs3102735 C>T	*OPG* rs2073618 G>C	*RANK* rs1805034 T>C	*RANKL* rs9533156 T>C	*RANKL* rs2277438 A>G
Chromosome	8	8	18	13	13
Gene Official Symbol	TNFRSF11B	TNFRSF11B	TNFRSF11A	TNFSF11	TNFSF11
Function	nearGene-5	missense	missense	missense	intron region
Chr Pos (Genome Build 36.3)	120034251	120033233	58178221	42045671	42053168
Regulome DB Score^a^	5	4	5	5	No Data
TFBS^b^	Y	—	—	—	—
Splicing (ESE or ESS)	—	Y	Y	—	—
miRNA (miRanda)	—	—	—	—	—
miRNA (Sanger)	—	—	—	—	—
MAF^c^ for Chinese in database	0.134	0.308	0.300	0.439	0.300
MAF in our controls (n = 686)	0.164	0.263	0.286	0.464	0.314
*p* value for HWE^d^ test in our controls	0.191	0.371	0.531	0.488	0.700
Genotyping method^e^	LDR	LDR	LDR	LDR	LDR
% Genotyping value	95.13%	96.35%	96.43%	96.43%	96.81%

a http://www.regulomedb.org/;

b TFBS: Transcription Factor Binding Site (http://snpinfo.niehs.nih.gov/snpinfo/snpfunc.htm);

c MAF: minor allele frequency, *OPG* rs2073618 G>C MAF is in CHB+JPT population;

d HWE: Hardy–Weinberg equilibrium;

e LDR: Ligation Detection Reaction.

### Associations between *OPG* rs3102735 C>T, *OPG* rs2073618 G>C, *RANK* rs1805034 T>C, *RANKL* rs9533156 T>C, *RANKL* rs2277438 A>G polymorphisms and risk of ESCC

Table 3 summarizes the genotype distribution of all five SNP polymorphisms in cases and controls. In the single locus analyses, the genotype frequencies of *RANK* rs1805034 T>C were 45.9% (TT), 42.9% (TC) and 11.2% (CC) in the case patients and 50.5% (TT), 41.8% (TC) and 7.7% (CC) in the control subjects. In the recessive model, when the *RANK* rs1805034 TT/TC genotypes were used as the reference group, the CC homozygote genotype was associated with a significantly increased risk for ESCC (CC vs. TT/TC: adjusted OR  = 1.52, 95% CI  = 1.03–2.24, *p* = 0.036). When the *RANK* rs1805034 TT homozygote genotype was used as the reference group, the TC genotype was not associated with the risk of ESCC (TC vs. TT: adjusted OR  = 1.16, 95% CI  = 1.03–2.24, *p* = 0.231), but the CC genotype was associated with the risk of ECSS (CC vs. TT: adjusted OR  = 1.62, 95% CI  = 1.08–2.44, *p* = 0.019). In the dominant model, the *RANK* rs1805034 CC variant was associated with the risk of ESCC as compared with the TT genotype (adjusted OR  = 1.62, 95% CI  = 1.08–2.44, *p* = 0.019).

**Table 3:** Logistic regression analyses of associations between *OPG* rs3102735 T>C, rs2073618 G>C, *RANK* rs1805034 T>C, *RANKL* rs9533156 T>C and rs2277438 A>G polymorphisms and risk of ESCC.

Genotype	Cases (n = 629)		Controls (n = 686)		Crude OR (95%CI)	*p*	Adjusted OR a (95%CI)	*p*
	n	%	n	%				
*OPG* rs3102735 T>C								
TT	442	73.7	450	69.1	1.00		1.00	
TC	146	24.3	188	28.9	0.79 (0.61–1.02)	0.069	0.78 (0.61–1.02)	0.065
CC	12	2.0	13	2.0	0.94 (0.42–2.08)	0.878	0.97 (0.43–2.19)	0.945
TC+CC	158	26.3	201	30.9	0.80 (0.63–1.02)	0.076	0.80 (0.62–1.02)	0.075
TT+TC	588	98.0	638	98.0	1.00		1.00	
CC	12	2.0	13	2.0	1.00 (0.45–2.21)	0.997	1.04 (0.46–2.34)	0.928
T allele	1030	85.8	1088	83.6	1.00			
C allele	170	14.2	214	16.4	0.84 (0.67–1.04)	0.116		
*OPG* rs2073618 G>C								
GG	345	56.6	361	54.9	1.00		1.00	
GC	222	36.4	246	37.4	0.94 (0.75–1.19)	0.631	0.96 (0.75–1.21)	0.702
CC	43	7.0	50	7.6	0.90 (0.58–1.39)	0.634	0.85 (0.55–1.32)	0.476
GC+CC	265	43.4	296	45.1	0.94 (0.75–1.17)	0.564	0.94 (0.75–1.17)	0.570
GG+GC	567	93.0	607	92.4	1.00		1.00	
CC	43	7.0	50	7.6	0.92 (0.60–1.41)	0.703	0.87 (0.56–1.34)	0.518
G allele	912	74.8	968	73.7	1.00			
C allele	308	25.2	346	26.3	0.95 (0.79–1.13)	0.533		
*RANK* rs1805034 T>C								
TT	282	45.9	330	50.5	1.00		1.00	
TC	264	42.9	273	41.8	1.13 (0.90–1.43)	0.296	1.16 (0.91–1.47)	0.231
CC	69	11.2	50	7.7	**1.62 (1.09**–**2.40)**	**0.018**	**1.62 (1.08**–**2.44)**	**0.019**
TC+CC	333	54.1	323	49.5	1.21 (0.97–1.50)	0.096	1.23 (0.98–1.54)	0.073
TT+TC	546	88.8	603	92.3	1.00		1.00	
CC	69	11.2	50	7.7	**1.52 (1.04**–**2.23)**	**0.031**	**1.52 (1.03**–**2.24)**	**0.036**
T allele	828	67.3	933	71.4	1.00			
C allele	402	32.7	373	28.6	**1.21 (1.03**–**1.44)**	**0.024**		
*RANKL* rs9533156 T>C								
TT	175	28.5	192	29.4	1.00		1.00	
TC	305	49.6	316	48.4	1.04 (0.78–1.38)	0.803	1.07 (0.80–1.42)	0.656
CC	135	22.0	145	22.2	0.98 (0.72–1.34)	0.894	1.06 (0.77–1.46)	0.708
TC+CC	440	71.5	461	70.6	1.02 (0.78–1.32)	0.913	1.07 (0.81–1.40)	0.645
TT+TC	480	78.0	508	77.8	1.00		1.00	
CC	135	22.0	145	22.2	0.96 (0.75–1.22)	0.710	1.02 (0.79–1.30)	0.903
T allele	655	53.3	700	53.6	1.00			
C allele	575	46.7	606	46.4	1.01 (0.87–1.19)	0.861		
*RANKL* rs2277438 A>G								
AA	277	46.2	315	46.8	1.00		1.00	
AG	259	43.2	294	43.7	1.00 (0.79–1.26)	0.988	1.00 (0.79–1.26)	0.981
GG	64	10.7	64	9.5	1.14 (0.78–1.67)	0.509	1.19 (0.80–1.75)	0.393
AG+GG	323	53.8	358	53.2	1.03 (0.82–1.28)	0.820	1.03 (0.82–1.29)	0.796
AA+AG	536	89.3	609	90.5	1.00		1.00	
GG	64	10.7	64	9.5	1.14 (0.79–1.64)	0.493	1.19 (0.82–1.73)	0.367
A allele	813	67.8	924	68.6	1.00			
G allele	387	32.3	422	31.4	1.04 (0.88–1.23)	0.627		

a Adjusted for age, sex, smoking status and alcohol consumption.

No association was detected among *OPG* rs3102735 C>T, *OPG* rs2073618 G>C, *RANKL* rs9533156 T>C, *RANKL* rs2277438 A>G polymorphisms and the risk of ECSS (Table 3).

### Stratification analyses of *RANK* rs1805034 T>C genotype and risk of ESCC

To evaluate the effects of *RANK* rs1805034 T>C genotype on ESCC risk according to different age, sex, smoking and alcohol consumption; we performed the stratification analyses (Table 4). A significantly increased risk of ESCC associated with the *RANK* rs1805034 T>C polymorphism was evident among male patients (CC vs. TT: adjusted OR  = 1.89, 95% CI  = 1.16–3.08, *p* = 0.011) (TC/CC vs. TT, adjusted OR  = 1.38, 95% CI  = 1.05–1.81, *p* = 0.022) (CC vs. TT/TC, adjusted OR  = 1.68, 95% CI  = 1.05–2.69, *p* = 0.031). Likewise, in elder patients (≥63 years old), *RANK* rs1805034 T>C polymorphism was also associated with a significantly increased risk of ESCC (CC vs. TT, adjusted OR  = 1.84, 95% CI  = 1.02–3.31, *p* = 0.041) (Table 4).

**Table 4:** Stratified analyses between *RANK* rs1805034 T>C polymorphism and ESCC risk by sex, age, smoking status and alcohol consumption.

Variable	*RANK* rs1805034 T>C (case/control) a				Adjusted OR b (95% CI); *p*; *p*_h_ c				
	TT	TC	CC	TC+CC	TT	TC	CC	TC+CC	CC vs. (TT+TC)
Sex									
Male	193/227	189/178	52/33	241/211	1.00	1.28 (0.96–1.71); *p*: 0.090; *p*_h_:0.221	**1.89 (1.16–3.08); ** ***p*** **: 0.011;** *p*_h_:0.290	**1.38 (1.05–1.81); ** ***p*** **: 0.022;** *p*_h_:0.155	**1.68 (1.05–2.69); ** ***p*** **: 0.031;** *p*_h_:0.448
Female	89/103	75/95	17/17	92/112	1.00	0.93 (0.62–1.42); *p*: 0.747; *p*_h_:0.221	1.22 (0.59–2.55); *p*: 0.594; *p*_h_:0.290	0.98 (0.66–1.46); *p*: 0.906; *p*_h_:0.155	1.26 (0.62–2.56); *p*: 0.520; *p*_h_:0.448
Age									
<63	131/161	136/152	34/28	170/180	1.00	1.11 (0.79–1.55); *p*: 0.563; *p*_h_:0.756	1.48 (0.83–2.61); *p*: 0.183; *p*_h_:0.664	1.16 (0.84–1.61); *p*: 0.360; *p*_h_:0.676	1.40 (0.81–2.42); *p*: 0.224; *p*_h_:0.702
≥63	151/169	128/121	35/22	163/143	1.00	1.18 (0.84–1.65); *p*: 0.338; *p*_h_:0.756	**1.84 (1.02–3.31); ** ***p*** **: 0.041;** *p*_h_:0.664	1.28 (0.93–1.76); *p*: 0.130; *p*_h_:0.676	1.71 (0.97–3.03); *p*: 0.063; *p*_h_:0.702
Smoking status									
Never	159/234	149/204	36/37	185/241	1.00	1.06 (0.79–1.43); *p*: 0.689; *p*_h_:0.451	1.37 (0.82–2.30); *p*: 0.228; *p*_h_:0.457	1.11 (0.84–1.47); *p*: 0.471; *p*_h_:0.358	1.33 (0.81–2.19); *p*: 0.255; *p*_h_:0.568
Ever	123/96	115/69	33/13	148/82	1.00	1.22 (0.81–1.84); *p*: 0.344; *p*_h_:0.451	1.89 (0.93–3.84); *p*: 0.080; *p*_h_:0.457	1.32 (0.90–1.95); *p*: 0.157; *p*_h_:0.358	1.73 (0.87–3.43);*p*: 0.119; *p*_h_:0.568
Alcohol consumption									
Never	190/248	180/215	45/38	225/253	1.00	1.09 (0.82–1.45); *p*: 0.551; *p*_h_:0.534	1.52 (0.93–2.48); *p*: 0.095; *p*_h_:0.753	1.16 (0.88–1.51); *p*: 0.298; *p*_h_:0.504	1.46 (0.91–2.33); *p*: 0.117; *p*_h_:0.871
Ever	92/82	84/58	24/12	108/70	1.00	1.16 (0.73–1.84); *p*: 0.530; *p*_h_:0.534	1.65 (0.76–3.60); *p*: 0.210; *p*_h_:0.753	1.24 (0.80–1.92); *p*: 0.332; *p*_h_:0.504	1.54 (0.73–3.28); *p*: 0.259; *p*_h_:0.871

a The genotyping was successful in 615 (97.8%) ESCC cases, and 653 (95.2%) controls for *RANK* rs1805034 T>C;

b Adjusted for age, sex, smoking status and alcohol consumption (besides stratified factors accordingly) in a logistic regression model;

c *p*_h_ for heterogeneity.

## Discussion

In this hospital-based case-control study of ESCC, we investigated the association of *OPG* rs3102735 C>T, *OPG* rs2073618 G>C, *RANK* rs1805034 T>C, *RANKL* rs9533156 T>C and *RANKL* rs2277438 A>G polymorphisms with risk of ESCC in a Chinese population. Our multivariable logistic analyses demonstrated that *RANK* rs1805034 T>C genotype has an increased risk of ESCC. Significant association with increased risk of ESCC was noticed among male patients and elder patients (≥63 years old). To our knowledge, this is the first study demonstrating a significant association between the *RANK* rs1805034 T>C genotype with the susceptibility of ESCC.

OPG was initially derived from an expressed sequence tag of a fetal rat intestine cDNA library encoding a 401-amino-acid polypeptide [11]. Subsequently, a physiological role of OPG in the maintenance of normal bone mass was underscored by several studies [11], [35], [36]. The later finding in murine myelomonocytic cell line 32D led to the identification of OPG binding protein or OPGL, which has identical sequence as RANKL and was further implicated with the osteoclast development [12]. Direct sequencing of a human bone marrow-derived myeloid dendritic cell cDNA library identified RANK as a novel TNFR homologue [13]. Subsequently, RANKL was identified from murine thymoma cell line EL40.5 [13] as well as in T cells [37]. RANKL exists as a homotrimer and induces receptor clustering upon engaging RANK on the cell surface, consequently causes receptor clustering. Activation events within the cell are initiated through TNFR-associated factors following sufficient RANK clustering. Genetic variants in the OPG locus have previously been implicated with osteoporotic fracture [38], bone turnover [39], bone mineral density [40], osteonecrosis [41], diabetic neuroarthropathy [42] as well as ankylosing spondylitis [43]. Alterations at the *RANK* locus and/or functionally related genes, such as *RANKL*, have also been reported to be associated with rheumatoid arthritis [30], aortic calcification [44], bone mineral density [39] and Paget's disease of bone [31]. Recently, emerging evidence has indicated an association between *OPG*/*RANK*/*RANKL* gene polymorphisms with carcinogenesis. Several studies demonstrated additional loci to be associated with breast cancer including the chromosomal region 8q24 for *OPG* gene [45], [46]. SNP rs3102735 of the *OPG* gene has been reported to be associated with the susceptibility of breast cancer in Caucasian population [10]. Similarly, a genetic variant near the 5′-end of *RANK* (rs7226991) was associated with a breast cancer risk [47]. The mechanism underlying the association remains obscure so far. Yet, vast majority of the association on chromosome 8q24 lies at approximately 128 Mb and is related to various tumor entities in addition to breast cancer, including prostate [48] and colon cancer [49].

Among different ethnic cohorts, the frequencies of genetic polymorphisms vary drastically. Our study demonstrated that the frequency of *RANK* rs1805034 C was 0.286 among 686 control subjects in Chinese population, which is lower than that of European (0.438) and African American (0.478), but similar with the Japanese population (0.311). However, interestingly, another study reported the frequency of *RANK* rs1805034 C was 0.476 in Han population from North China, which differs our finding in cohort from East China, suggesting the ethnical impact could also be interfered with regional environmental factors (http://www.ncbi.nlm.nih.gov/projects/SNP/snp_ref.cgi? rs = 1805034). Using Power and Sample Size Calculation (PS, version 3.0.43, 2009, http://biostat.mc.vanderbilt.edu/twiki/bin/view/Main/PowerSampleSize), considering *RANK* rs1805034 T>C mutant alleles, the power of our analysis (α = 0.05) was 0.946 in 629 ESCC cases and 686 control subjects with adjusted OR 1.52. In male subgroup, the power of our analysis was 0.995 among 434 cases and 438 control subjects, with the adjusted OR 1.89. In elder cohort (≥63), the power of analysis (α = 0.05) was 0.962 among 314 cases and 312 controls with adjusted OR 1.84. The current study has revealed the association with increased risk of ESCC among male patients and elder patients (≥63 years old), which was in consistent with the previous report. In a retrospective study involving 74,854 ESCC patients from North China, the prevalence among males was higher than that among females, similar to our findings. Moreover, this study demonstrated that although the prevalence significantly declined, the median age-of-onset of ESCC postponed [50], verifying our notion that elder population has higher risk.

In conclusion, our study provides with the evidence that functional polymorphism of *RANK* rs1805034 T>C is associated with the susceptibility of ESCC. We acknowledge there are several limitations in this study that need to be addressed. First of all, the study subjects were all recruited from several local medical centers within same area, which may not completely represent the general Chinese population, especially when diverse regional environmental factors exist. Secondly, the detailed information regarding cancer metastasis and survival were not provided as the follow-up study is still ongoing, which hinders further analyses of the impact of these SNP polymorphisms on the ESCC progression and prognosis. Lastly, as the epidemiologic complexities of esophageal cancer are vast, rendering screening and prevention limited at best. The association between nutrition factors, exposure to fungal toxins or N-nitroso-compound in food and risk of ESCC is not studied. Further studies among different regions or ethnic populations with diverse nutrition conditions, and supplemented with functional analyses, are warranted to verify our findings.
